# Inhibition of CaMKIIα Activity Enhances Antitumor Effect of Fullerene C60 Nanocrystals by Suppression of Autophagic Degradation

**DOI:** 10.1002/advs.201801233

**Published:** 2019-02-10

**Authors:** Jing Xu, Hongsheng Wang, Yi Hu, Yu Shrike Zhang, Longping Wen, Fei Yin, Zhuoying Wang, Yingchao Zhang, Suoyuan Li, Yanyan Miao, Binhui Lin, Dongqing Zuo, Gangyang Wang, Min Mao, Tao Zhang, Jianxun Ding, Yingqi Hua, Zhengdong Cai

**Affiliations:** ^1^ Department of Orthopedics Shanghai General Hospital Shanghai Jiao Tong University School of Medicine Shanghai Bone Tumor Institution 100 Haining Street Shanghai 200080 P. R. China; ^2^ Shanghai Bone Tumor Institution 100 Haining Street Shanghai 200080 P. R. China; ^3^ Hefei National Laboratory for Physical Sciences at the Microscale and School of Life Sciences University of Science and Technology of China 96 Jinzhai Street Hefei 230026 P. R. China; ^4^ Division of Engineering in Medicine Department of Medicine Brigham and Women's Hospital Harvard Medical School 65 Landsdowne Street Cambridge MA 02139 USA; ^5^ School of Medicine South China University of Technology Nanobio Laboratory Institutes for Life Sciences South China University of Technology 381 Wushan Street Guangzhou 510006 P. R. China; ^6^ Key Laboratory of Gene Engineering of the Ministry of Education State Key Laboratory of Biocontrol School of Life Sciences Sun Yat‐sen University 135 West Xingang Street Guangzhou 510275 P. R. China; ^7^ Key Laboratory of Polymer Ecomaterials Changchun Institute of Applied Chemistry Chinese Academy of Sciences 5625 Renmin Street Changchun 130022 P. R. China

**Keywords:** autophagic degradation, autophagy, calcium/calmodulin‐dependent protein kinase IIα, fullerene C60 nanocrystals, osteosarcoma therapy

## Abstract

Fullerene C60 nanocrystals (nano‐C60) possess various attractive bioactivities, including autophagy induction and calcium/calmodulin‐dependent protein kinase IIα (CaMKIIα) activation. CaMKIIα is a multifunctional protein kinase involved in many cellular processes including tumor progression; however, the biological effects of CaMKIIα activity modulated by nano‐C60 in tumors have not been reported, and the relationship between CaMKIIα activity and autophagic degradation remains unclear. Herein, nano‐C60 is demonstrated to elicit reactive oxygen species (ROS)‐dependent cytotoxicity and persistent activation of CaMKIIα in osteosarcoma (OS) cells. CaMKIIα activation, in turn, produces a protective effect against cytotoxicity from nano‐C60 itself. Inhibition of CaMKIIα activity by either the chemical inhibitor KN‐93 or CaMKIIα knockdown dramatically promotes the anti‐OS effect of nano‐C60. Moreover, inhibition of CaMKIIα activity causes lysosomal alkalinization and enlargement, and impairs the degradation function of lysosomes, leading to autophagosome accumulation. Importantly, excessive autophagosome accumulation and autophagic degradation blocking are shown to play an important role in KN‐93‐enhanced‐OS cell death. The synergistic anti‐OS efficacy of KN‐93 and nano‐C60 is further revealed in an OS‐xenografted murine model. The results demonstrate that CaMKIIα inhibition, along with the suppression of autophagic degradation, presents a promising strategy for improving the antitumor efficacy of nano‐C60.

## 1 Introduction

In a wide range of nanoscale structures, inorganic nanocrystals (e.g., quantum dots, fullerene, gold, iron oxide, silica, and others) have been extensively explored as potential therapeutic systems in the field of oncology.^1^, ^2^, ^3^, ^4^, ^5^ Among these, fullerene C60 is of particular interest for cancer therapy due to its unique geometrical structure and remarkable physicochemical properties.^6^ The predominant use of fullerene C60 for tumor treatment has been as an antitumor reagent^7^, ^8^, ^9^ or nanocarrier.^10^, ^11^ However, to date, our understanding regarding the antitumor biological effects that fullerenes possess remains limited.

In our previous work, we found that the water‐suspended fullerene C60 nanocrystals (nano‐C60) specifically bind to the hippocampal Ca^2+^ signaling protein, CaMKIIα. This interaction causes persistent CaMKIIα activation, as well as increased learning and memory of rats.^12^, ^13^ CaMKII (Ca^2+^/calmodulin‐dependent protein kinase II) is an important intracellular serine/threonine kinase, mainly expressed in neuronal cells. In addition to nerve signal transduction, CaMKII also plays an essential role in regulating tumor cell survival, proliferation, and differentiation.^14^, ^15^, ^16^, ^17^ Nevertheless, the biological effects of nano‐C60 on CaMKIIα in tumors and the role of CaMKIIα in the antitumor activity of nano‐C60 have yet to be reported.

Several studies have documented remarkable antitumor effects of fullerenes via a variety of mechanisms involving oxidative stress,^18^ antiangiogenesis,^19^ immunomodulation,^20^ and autophagy modulation.^21^ Autophagy is a lysosomal degradation pathway in eukaryotic cells, by which unnecessary or dysfunctional cellular components are sequestered into double‐membrane vesicles (autophagosomes). Then autophagosomes fuse with lysosomes to degrade and recycle the sequestered material.^22^ Autophagy has been proven to be important for intracellular quality control and cell fate‐regulating processes, including cell survival during stress^22^ and cell death.^23^ As autophagy is a cellular degradation pathway, the primary physiological functions of autophagy mainly depend on its degradation capacity.^24^ For example, when cells are under stress, autophagy levels are actively elevated. This is followed by upstream autophagosome formation and downstream degradation reaching a dynamic balance state. However, if overstimulation of autophagy is maintained at a relatively high level, excessive consumption through autophagic degradation may cause cell death.^[23]^ Alternatively, blocking or disrupting autophagy degradation at a downstream step can lead to a large increase in the accumulation of autophagic vacuoles, which may also be catastrophic for cells.^25^, ^26^ Therefore, modulating autophagy via autophagy induction or autophagic flux blockage has been associated with different physiological consequences, which is a promising strategy for tumor therapy.

Nano‐C60 and its derivatives have been found to sensitize cancer cells to chemotherapeutic agents by inducing autophagy.^21^, ^27^ Although these studies showed that fullerenes induce autophagosome accumulation, autophagic flux was not investigated. Moreover, the relationship between CaMKIIα activity and autophagy degradation, and the influence of these two events on nano‐C60‐mediated cytotoxicity have not been reported.

Osteosarcoma (OS) is a primary malignant bone tumor with a high propensity of invasion and metastasis, mainly occurring in children and adolescents. Despite improvements in surgery and multi‐agent chemotherapy, the 5‐year survival rate of patients with OS is only approximately 65% and has remained largely unchanged over the past three decades.^28^ Several studies have proven the key role of CaMKIIα in regulating OS progression and metastasis.^29^, ^30^ Nevertheless, the underlying mechanism of CaMKIIα function in OS has not been adequately clarified.

**Scheme 1:**
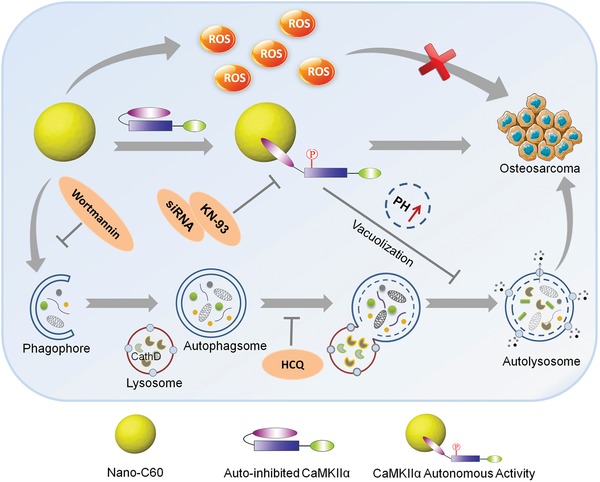
Schematic illustration of combination treatment strategy of nano‐C60 and CaMKIIα inhibition for OS therapy.

In this report, the antitumor activity of nano‐C60 in OS cells with respect to CaMKIIα was investigated, as illustrated in **Scheme** **1**. Nano‐C60 has different biological effects on OS cells, including ROS production, CaMKIIα activation, and autophagy induction. First, nano‐C60 elicits cytotoxicity in OS cells via ROS production. Second, nano‐C60 induces abnormal autophagy, which leads to autophagosome accumulation. Third, nano‐C60 causes cytoprotective autonomous CaMKIIα activity against OS cell death. Pharmacological or genetic inhibition of CaMKIIα enhances the anti‐OS effect of nano‐C60. Furthermore, this study demonstrates for the first time that CaMKIIα inhibition is involved in autophagy modulation, which enhances excessive autophagosome accumulation and autophagy degradation blockage through lysosomal alkalinization, enlargement, and dysfunction, and thus leads to increased nano‐C60 cytotoxicity. Therefore, these results suggest that CaMKIIα activity should be considered when employing nano‐C60 for tumor therapy. This study provides a novel strategy for the use of CaMKIIα activity and autophagic degradation as therapeutic targets to improve the efficacy of nano‐C60 in tumor treatment.

## 2 Results

### 2.1 Nano‐C60 Induces ROS‐Dependent Cytotoxicity

Water‐suspended nano‐C60 was prepared using a standard tetrahydrofuran evaporation procedure,^21^ and the particles exhibited mainly circular and rectangular morphologies with an average size of 120 nm, as revealed by transmission electron microscopy (Figure S1, Supporting Information). We first evaluated the cytotoxicity of nano‐C60 in a panel of OS cells (143B, MG63, Saos_2_, SJSA, and HOS cells; Figure S2A, Supporting Information).

Nano‐C60 is known to produce ROS.^18^, ^21^ Consistent with this property, our preparation of nano‐C60 generated ROS in 143B and MG63 cells, as detected by dihydroethidium staining (Figure S2C, Supporting Information). Reduced glutathione as a free radical scavenger could effectively reduce the cytotoxicity induced by nano‐C60 (Figure S2B, Supporting Information), indicating that nano‐C60 induced ROS‐dependent cytotoxicity in OS cells.

### 2.2 Nano‐C60 Elicits CaMKIIα Autonomous Activity in OS Cells

Our previous report demonstrated that nano‐C60‐CaMKIIα interaction elicits rat hippocampal CaMKIIα autonomous activity.^12^ CaMKII is a multimeric protein usually composed of 12 subunits. Each subunit comprises three domains, an N‐terminal catalytic domain for exerting catalytic function, a regulatory domain for controlling kinase activation, and a C‐terminal association domain for mediating multimerization.^31^ Under resting conditions, the autoinhibitory region in the CaMKII regulatory domain interacts with the catalytic domain, which blocks the substrate binding sites and thereby inhibits CaMKII kinase activity. When intracellular Ca^2+^ levels rise, Ca^2+^/calmodulin (Ca^2+^/CaM) binds to the regulatory domain of CaMKII, causing the closed CaMKII conformation to open and the enzyme to become active. This activation triggers the phosphorylation of adjacent CaMKII subunits at residues T286/T287 within the autoinhibitory region, leading to Ca^2+^/CaM‐independent activity, namely, autonomous activity.^31^

**Figure 1:**
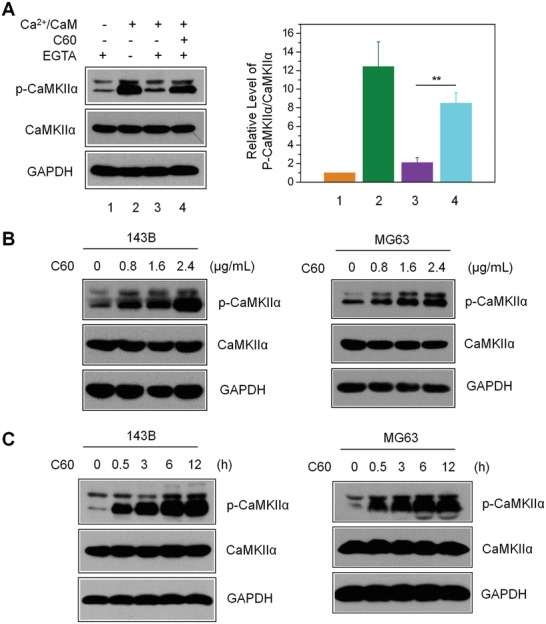
Nano‐C60‐induced autonomous CaMKIIα activity in OS cells. A) T286 autophosphorylation assay for CaMKIIα in 143B cell lysates, as detected by anti‐CaMKII and phospho‐CaMKII antibodies. The right panel shows the level of p‐CaMKIIα relative to total CaMKIIα, with the value for control (without Ca^2+^/CaM and nano‐C60) set at 1. Mean ± SEM, *n* = 3. **P < 0.01. B) Dose‐dependent CaMKIIα‐T286 autophosphorylation level in 143B and MG63 cells treated with nano‐C60 for 12 h. C) Time course of CaMKIIα‐T286 autophosphorylation levels in 143B and MG63 cells treated with 2.4 µg mL^−1^ nano‐C60.

As CaMKIIα is highly conserved among species, we assessed the impact of nano‐C60 on CaMKIIα activity in OS cells. First, we conducted in vitro assays to detect T286 phosphorylation of CaMKIIα by using 143B cell lysates. Basal phosphorylation levels were very low but exhibited a 12‐fold increase under Ca^2+^/CaM treatment. This stimulated phosphorylation was significantly abolished after depleting intracellular Ca^2+^ by EGTA treatment (**Figure** **1**A). In comparison, the addition of nano‐C60 sustained the Ca^2+^/CaM‐stimulated phosphorylation even in the presence of EGTA, suggesting that nano‐C60‐induced phosphorylation was independent of sustained Ca^2+^/CaM treatment. This was similar to the effect of adenosine triphosphate (ATP)‐induced CaMKIIα T286 autophosphorylation. Next, the nano‐C60‐induced T286 phosphorylation in OS cells detected by Western blot was both time (Figure 1C) and dose dependent (Figure 1B). These results indicated that nano‐C60 enhanced the autonomous activity of CaMKIIα in OS cells.

### 2.3 Inhibition of CaMKIIα Activity Enhances Nano‐C60‐Induced Cytotoxicity

**Figure 2:**
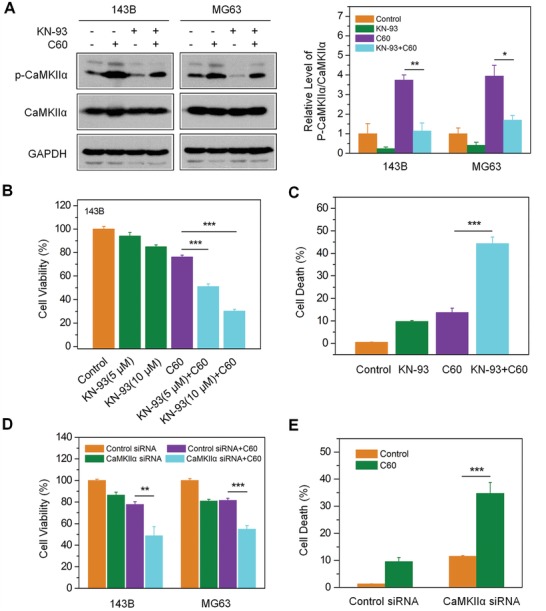
Effects of CaMKIIα inhibition on nano‐C60‐induced cytotoxicity in OS cells. A)143B and MG63 cells were treated with 1.6 µg/mL^−1^ of nano‐C60 in the presence or absence of 10.0 × 10^−6^ m KN‐93 for 24 h. CaMKIIα level was detected by Western blotting with antibodies against CaMKII and phospho‐CaMKII. The right panel demonstrates the level of p‐CaMKIIα relative to that of total CaMKIIα, with the control value (without nano‐C60) set at 1. Mean ± SEM, *n* = 3. *P < 0.05, **P < 0.01. B) 143B cells were treated with or without 1.6 µg mL^−1^ of nano‐C60 in the presence or absence of 5.0 or 10.0 × 10^−6^ m KN‐93 for 24 h. Cell viability was measured by CCK‐8 assay. Mean ± SEM, *n* = 3. ***P < 0.005. C) Cell death assay of 143B cells treated as in A). Cell death rates were determined by Hoechst/PI staining and demonstrated as the percentage of PI‐positive cells. Mean ± SEM, *n* = 3. ***P < 0.005. D) Cell viability of 143B and MG63 cells treated with or without 1.6 µg mL^−1^ of nano‐C60 for 24 h after transfection with CaMKIIα siRNA or control siRNA for 48 h. Mean ± SEM, *n* = 3. **P < 0.01, ***P < 0.005. E) The cell death rates of 143B cells treated as described in D). Mean ± SEM, *n* = 3. ***P < 0.005.

CaMKIIα activation has been suggested to promote cell proliferation, invasion, and metastasis in OS.^29^, ^30^ To evaluate the role of CaMKIIα in nano‐C60‐induced cytotoxicity, we employed KN‐93, the most extensively used inhibitor for studying in vitro and in vivo functions of CaMKII.^32^ As shown in **Figure** **2**A, KN‐93 significantly inhibited nano‐C60‐induced phosphorylation of CaMKIIα in 143B and MG63 cells. Compared to nano‐C60 treatment alone, pretreatment of cells with KN‐93 further decreased 143B cell viability by approximately 25.13% (5 × 10^−6^ m KN‐93) and 46.11% (10 × 10^−6^ m KN‐93) (Figure 2B). Similar results were observed in MG63 cells (Figure S3, Supporting Information). The cell death rate of 143B cells detected by Hoechst 33 342/propidium iodide (PI) staining demonstrated that KN‐93 enhanced nano‐C60‐induced 143B cell death by 30.55% (Figure 2C). These results demonstrated that combining KN‐93 and nano‐C60 treatments had a significant synergistic effect in OS cells.

To further confirm the role of CaMKIIα in nano‐C60‐treated OS cells, we employed siRNA to silence CaMKIIα protein expression (Figure S4, Supporting Information). Compared to the control siRNA group, 143B cells transfected with CaMKIIα‐specific siRNA followed by nano‐C60 treatment exhibited a distinct decrease in cell viability (Figure 2D) and an increase in cell death (Figure 2E).

Collectively, the results above demonstrated that nano‐C60‐induced CaMKIIα activity played a protective role in OS cell fate. Inhibition of CaMKIIα activity by either the chemical inhibitor KN‐93 or by CaMKIIα knockdown enhanced the cytotoxicity of nano‐C60 in OS cells.

### 2.4 Inhibition of CaMKIIα Activity Promotes Nano‐C60‐Induced Autophagosome Accumulation and Impairs Autophagic Degradation

**Figure 3:**
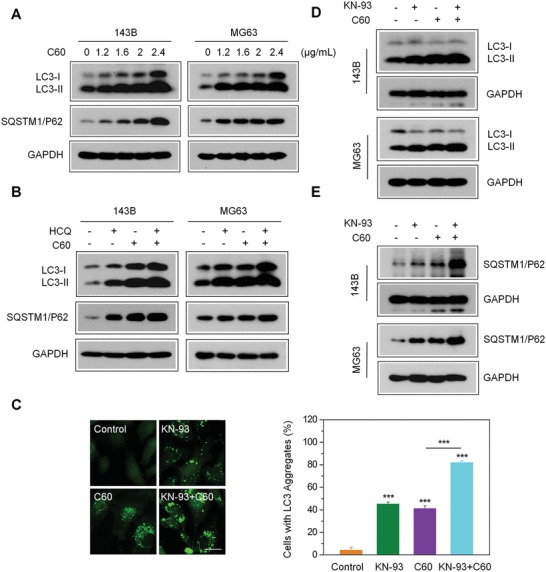
Enhancement of nano‐C60‐induced incomplete autophagy by CaMKIIα inhibition. A) Western blotting for the expression of autophagy‐associated proteins in 143B and MG63 cells treated with different doses of nano‐C60 for 24 h using antibodies against LC3, SQATM1/P62, and GAPDH. B) 143B cells were treated with or without 1.6 µg mL^−1^ nano‐C60 in the presence or absence of 100.0 × 10^−6^ m HCQ for 24 h. LC3 and SQATM1/P62 levels were examined by Western blotting with anti‐LC3 and anti‐SQATM1/P62 antibodies, respectively. C) Representative fluorescence microscopy images of MG63‐EGFP‐LC3 cells treated with or without 1.6 µg mL^−1^ of nano‐C60 in the presence or absence of 10.0 × 10^−6^ m KN‐93 for 24 h. Scale bar = 20.0 × 10^−6^ m . The right panel demonstrates the rate of EGFP‐LC3 dots‐positive cells. Mean ± SEM, *n* = 3. ***P < 0.005. D,E) Western blot analysis of the expression of LC3‐II and SQATM1/P62 in 143B and MG63 cells treated with or without 1.6 µg mL^−1^ of nano‐C60 in the presence or absence of 10.0 × 10^−6^ m KN‐93 for 24 h.

A previous report revealed that nano‐C60 induces autophagy and sensitizes cancer cells to chemotherapeutic killing,^21^ which inspired us to investigate the potential relationship between nano‐C60‐stimulated autophagy and CaMKIIα activity. As the conversion from soluble microtubule‐associated protein 1 light chain 3 (LC3‐I) to insoluble LC3 (LC3‐II) is a hallmark of autophagy,^33^ we used MG63 cells expressing enhanced green fluorescent protein‐tagged LC3 (MG63‐EGFP‐LC3) to assess the autophagy‐inducing ability of nano‐C60 in OS. The green fluorescence of EGFP‐LC3 was diffuse throughout the cytoplasm of control cells, while several bright green punctate structures accumulated in the nano‐C60‐treated cells (**Figure** **3**C). Moreover, the level of LC3‐II was significantly elevated in a dose‐dependent manner after nano‐C60 treatment, as shown by Western blot analysis (Figure 3A). These data suggested that nano‐C60 caused the accumulation of autophagosomes in OS cells.

As autophagy is a dynamic process, autophagosome accumulation may be due to the increase in autophagosome formation (autophagy induction) or the decrease in autophagosome degradation.^34^ To detect the autophagic flux induced by nano‐C60, we treated cells with hydroxychloroquine (HCQ), a classic autophagy inhibitor that disrupts autolysosome degradation by blocking vacuolar H^+^‐ATPase activity. The addition of HCQ significantly enhanced nano‐C60‐induced LC3‐II accumulation in both 143B and MG63 cells, indicating that nano‐C60 enhanced new autophagosome formation (Figure 3B). SQSTM1/P62, an LC3‐binding protein, is degraded via autophagy.^35^ Interestingly, the abundance of SQSTM1/P62 increased after nano‐C60 treatment (Figure 3A), and co‐treatment with HCQ and nano‐C60 further increased the level of SQSTM1/P62 (Figure 3B), which indicated a possible impairment of nano‐C60‐induced autophagy turnover.

Next, we examined the impact of CaMKIIα inhibition on nano‐C60‐induced autophagy. As shown in Figure 3C, KN‐93 markedly enhanced the formation of green punctate dots in nano‐C60‐treated cells, and Western blotting results showed an increased accumulation of LC3‐II (Figure 3D), but a decreased degradation of SQSTM1/P62 in these cells (Figure 3E). These results suggested that inhibition of CaMKIIα activity promoted nano‐C60‐induced autophagosome accumulation and reduced autophagic degradation in OS cells.

### 2.5 Nano‐C60 and KN‐93 Co‐Treatment Causes Lysosomal Alkalinization and Enlargement

**Figure 4:**
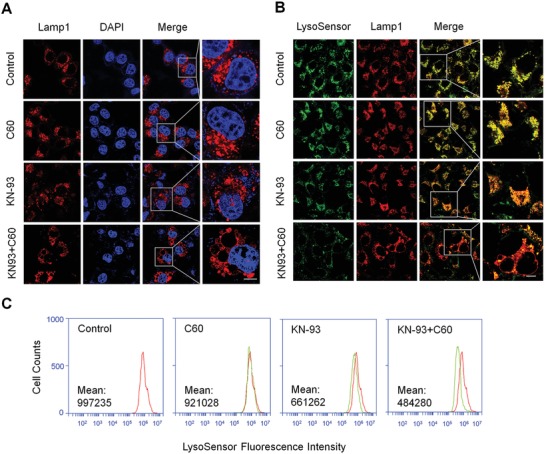
Lysosomal alkalinization and enlargement caused by nano‐C60 and KN‐93 combination treatment. A) Representative fluorescence images of 143B‐RFP‐Lamp1 cells treated with or without 1.6 µg mL^−1^ of nano‐C60 in the presence or absence of 10.0 × 10^−6^ m KN‐93 for 16 h. Nuclei were stained with DAPI (blue). Scale bar = 20.0 µm. B) Representative fluorescence images of lysosome acidity using LysoSensor Green DND‐189 staining in 143B‐RFP‐Lamp1 cells treated with or without 1.0 µg mL^−1^ of nano‐C60 in the presence or absence of 7.5 × 10^−6^ m KN‐93 for 12 h. Scale bar = 50.0 µm. C) Flow cytometric analysis of lysosome acidity in 143B cells treated as described in B).

Several types of inorganic nanoparticles, including gold nanoparticles^36^ and surface‐functionalized silica nanoparticles,^37^ have been reported to cause lysosomal dysfunction. As autophagy is a kind of lysosome‐based degradative pathway, disruption of lysosomal function or impairment of the lysosomal degradation capacity may block autophagic flux. Lysosomal‐associated membrane protein 1 (Lamp‐1) is a classic marker for lysosomal membranes. Confocal imaging of red fluorescence protein (RFP)‐Lamp‐1 in 143B cells revealed that nano‐C60 and KN‐93 co‐treatment promotes lysosomal enlargement and vacuolization (**Figure** **4**A), which likely represent a state of profound lysosomal stress and dysfunction.^38^, ^39^ It is well documented that defective lysosomal degradation induces lysosomal enlargement.^40^ The normal lysosomal degradation function depends on an acidic environment, and thus, we measured the acidity of lysosomes in 143B‐RFP‐Lamp1 cells with LysoSensor Green DND‐189, which is an acidotropic dye exhibiting the increase in the fluorescence intensity under acidification.^41^ To attenuate the influence of significant cytotoxicity induced by the combination treatment on lysosomal acidity, we used a relatively low dose (1.0 µg mL^−1^ of nano‐C60 and 7.5 × 10^−6^ m KN‐93 for 12 h). Fluorescence imaging (Figure 4B) and flow cytometric analysis (Figure 4C) showed that the fluorescence intensity of LysoSensor Green was slightly reduced in KN‐93‐treated cells but was dramatically reduced in cells co‐treated with KN‐93 and nano‐C60. Furthermore, LysoSensor Green was well colocalized with RFP‐Lamp1, which confirmed that the acidic compartments stained by LysoSensor Green were Lamp1 positive structures (lysosomes) (Figure 4B). These data indicated that the combination treatment caused alkalinization and enlargement of lysosomes.

### 2.6 Nano‐C60 and KN‐93 Co‐Treatment Impairs the Lysosomal Degradation Capacity

**Figure 5:**
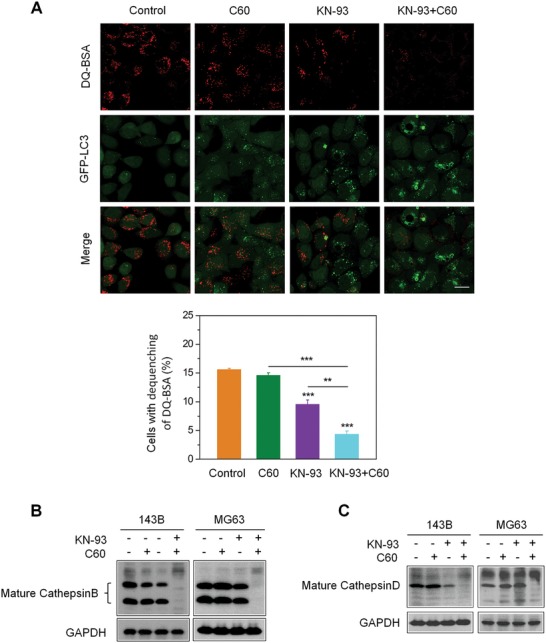
Impaired lysosomal degradation capacity caused by nano‐C60 and KN‐93 combination treatment. A) DQ‐BSA analysis of lysosomal proteolytic activity in 143B cells treated with or without 1.0 µg mL^−1^ of nano‐C60 in the presence or absence of 7.5 × 10^−6^ m KN‐93 for 12 h. The average number of the red fluorescent fragments in each cell was quantified (lower panel). At least 60 cells were analyzed for each treatment. Mean ± SEM, *n* = 3. **P < 0.01 ***P < 0.005. Scale bar = 50.0 µm. B) and C) Western blot analysis of enzymatic activity of cathepsin B and cathepsin D in 143B and MG63 cells treated with or without 1.6 µg mL^−1^ of nano‐C60 in the presence or absence of 10.0 × 10^−6^ m KN‐93 for 24 h.

To determine whether the combination treatment impairs the lysosomal degradation capacity, we pretreated MG63‐EGFP‐LC3 cells with a lysosomal degradation indicator, derivative‐quenched bovine serum albumin (DQ‐BSA).^42^ Enzymatic cleavage of DQ‐BSA in lysosomes results in the generation of fluorescent fragments. A large number of red fluorescent fragments (dequenching of DQ‐BSA) occurred in the control group. In contrast, very a few red fluorescent fragments occurred in the co‐treatment group (**Figure** **5**A), indicative of the impairment of lysosomal degradation capacity. Moreover, the dequenching of DQ‐BSA was negatively correlated with GFP‐LC3B puncta (Figure 5A), suggesting that the inhibition of lysosomal degradation resulted in the accumulation of autophagosomes.

Cathepsins (cathepsin B and cathepsin D), the classical lysosomal marker proteases, participate in autophagic degradation.^43^ Then, we detected the effect of the combination treatment on the lysosomal proteolytic activity. As shown in Figure 5B,C, the maturation levels of cathepsin B and cathepsin D in 143B and MG63 cells were dramatically reduced after co‐treatment.

Taken together, we speculated that nano‐C60 and KN‐93 co‐treatment promoted lysosomal alkalinization and impaired the lysosomal degradation capacity, leading to inhibition of autophagic degradation and autophagosome accumulation, and thus causing lysosomal vacuolization and enlargement.

### 2.7 Excessive Autophagosome Accumulation Contributes to the Cytotoxicity Elicited by Nano‐C60 and KN‐93 Combination Treatment

**Figure 6:**
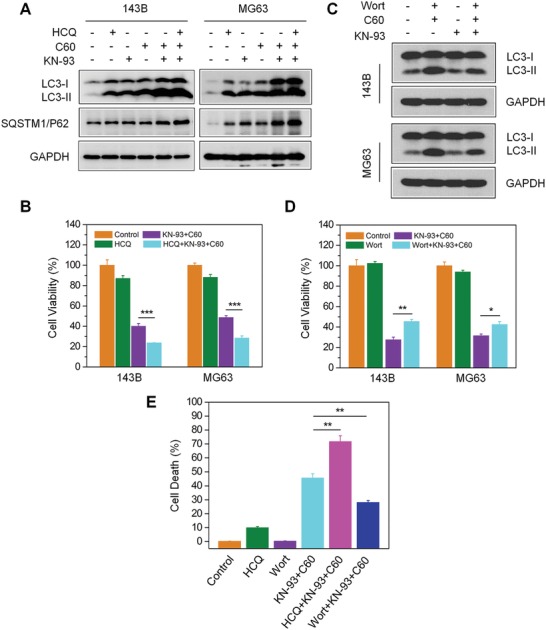
Effects of autophagosome accumulation on cytotoxicity induced by nano‐C60 and KN‐93 combination treatment. A,B) Before 1.6 µg mL^−1^ of nano‐C60 and 10.0 × 10^−6^ m KN‐93 addition, 143B cells were pretreated with or without 100.0 × 10^−6^ m HCQ. A) LC3 and SQATM1/P62 levels were detected by Western blotting using anti‐LC3 and anti‐SQATM1/P62 antibodies, respectively, and B) cell viability was assessed by CCK‐8 assay. Mean ± SEM, *n* = 3. ***P < 0.005. C) Before 1.6 µg mL^−1^ of nano‐C60 and 10 × 10^−6^ m KN‐93 addition, 143B and MG63 cells were pretreated with or without 100.0 × 10^−9^ m Wort. LC3 levels were detected by Western blotting using anti‐LC3 and anti‐SQATM1/P62 antibodies, respectively. D) Cell viability of each group treated as described in C) was assessed by CCK‐8 assay. Mean ± SEM, *n* = 3. *P < 0.05, **P < 0.01. E) Before nano‐C60 and KN‐93 addition, 143B cells were pretreated with or without HCQ or Wort. Cell death assay was determined by Hoechst/PI staining and demonstrated as the percentage of PI‐positive cells. Mean ± SEM, *n* = 3. **P < 0.01. Scale bar = 200.0 µm.

To evaluate the role of autophagic degradation in the cytotoxicity elicited by nano‐C60 and KN‐93 co‐treatment, we employed an upstream autophagy inhibitor, wortmannin (Wort), and a downstream autophagy inhibitor, HCQ. The addition of HCQ after nano‐C60 and KN‐93 co‐treatment dramatically increased LC3‐II and SQSTM1/P62 accumulation (**Figure** **6**A), decreased cell viability (Figure 6B), and enhanced cell death (Figure 6E). These results indicated that the accumulation of autophagosomes and the inhibition of autophagic degradation further enhanced the cytotoxicity elicited by the combination treatment.

Next, we alleviated autophagosome accumulation through an upstream autophagy inhibitor Wort, which blocks autophagosome formation via suppression of the class III phosphatidylinositol 3‐kinase (PtdIns3K) signaling pathway. Wort effectively inhibited LC3‐II conversion induced by nano‐C60 alone (Figure S5A, Supporting Information) or nano‐C60 and KN‐93 co‐treatment (Figure 6C), and decreased the cytotoxicity of nano‐C60 itself (Figure S5B, Supporting Information). Furthermore, pretreatment with Wort promoted cell viability (Figure 6D) and attenuated cell death (Figure 6E) after nano‐C60 and KN‐93 co‐treatment.

From these results, we concluded that autophagic degradation is involved in the nano‐C60 and KN‐93 combined treatment‐induced biological effects in OS cells. KN‐93 enhanced nano‐C60‐elicited cytotoxicity through autophagic degradation inhibition and the excessive autophagosome accumulation.

### 2.8 Inhibition of CaMKIIα Activity Enhances the Anti‐OS Efficacy of Nano‐C60 In Vivo

**Figure 7:**
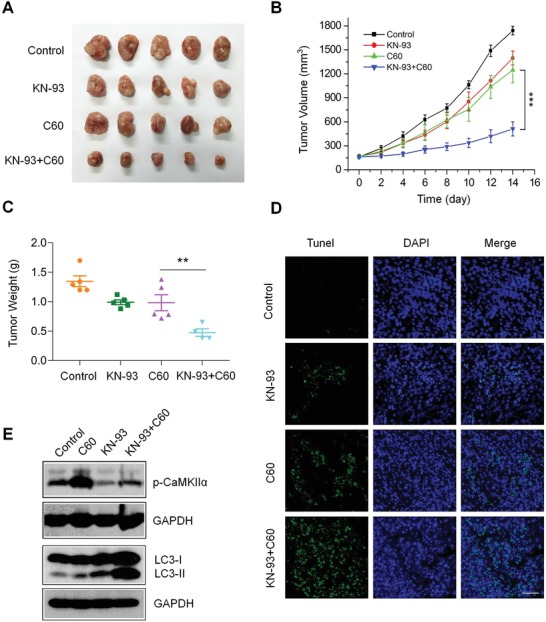
Enhanced synergistic anti‐OS efficacy of nano‐C60 by CaMKIIα inhibitor KN‐93 in vivo. A) Tumors from nude mice treated with PBS (control), 0.5 mg kg^−1^ KN‐93 (s.c.), 0.2 mg kg^−1^ nano‐C60 (s.c.), or 0.5 mg kg^−1^ KN‐93 plus 0.2 mg kg^−1^ nano‐C60 (s.c.). B) Tumor volume and C) tumor weight in each group are shown. Mean ± SEM, *n* = 5. **P < 0.01, ***P < 0.005. D) TUNEL staining (green) of tumor tissues was performed to detect apoptotic cells, and nuclei were treated with DAPI (blue). Scale bar = 50.0 µm. E) Western blot analysis of the levels of phospho‐ or total‐CaMKIIα, and LC3 in tumor tissues.

To determine the synergistic ablation effect of nano‐C60 and KN‐93 in vivo, a 143B‐derived subcutaneous tumor xenograft model was established. BALB/c nude mice were injected with 143B cells in the right armpit. On the 8th day postinjection, tumor‐bearing mice were randomly divided into four groups: PBS (control), KN‐93, nano‐C60, and KN‐93+nano‐C60. Mice were administrated a dose every other day, and tumor size and body weight were also measured. After two weeks of treatment, the mice were sacrificed, and their subcutaneous tumors were gently removed, photographed, and weighed. No significant changes in body weight were observed after any of the treatments (Figure S6, Supporting Information). As depicted in **Figure** **7**A,B, tumor volume results indicated that nano‐C60 alone and KN‐93 alone slightly inhibited tumor growth. However, in comparison to the single‐drug treatments, the combination of KN‐93 and nano‐C60 significantly suppressed tumor growth. Similar results were observed in tumor weight (Figure 7C). These results confirmed that combined treatment of nano‐C60 with KN‐93 had a significant synergistic effect in ablating OS.

Furthermore, terminal deoxyribonucleotidyl transferase (TDT)‐mediated dUTP‐digoxigenin nick end labeling (TUNEL) staining demonstrated that there were more apoptotic cells in the tumors of the co‐treatment group than in those of the single‐drug groups (Figure 7D), which was consistent with results from hematoxylin and eosin staining (Figure S7, Supporting Information). In addition, immunoblot analysis of the tumor tissues demonstrated that the expression of phosphorylated CaMKIIα was effectively inhibited, while the level of autophagy was significantly elevated after the combination treatment (Figure 7E). Collectively, the results above show that inhibition of CaMKIIα activity enhances the antitumor efficacy of nano‐C60 in OS.

## 3 Discussion

In this report, we demonstrated that nano‐C60 elicited ROS‐dependent anti‐OS activity. Moreover, the autonomous CaMKIIα activity induced by nano‐C60 played a protective role in OS cell fate and attenuated the anticancer effect of nano‐C60. KN‐93, the most widely used CaMKII inhibitor, has been found to cause cell cycle arrest or cell death in a variety of tumors.^44^ Few studies have reported the combination treatment of KN‐93 and anticancer agents, especially nanodrugs. In this study, we showed that in comparison to a single‐agent treatment at the same concentration, the combination of KN‐93 and nano‐C60 at a relatively low dose exerted outstanding synergistic anti‐OS effects in vitro and in vivo. These results supported the potential use of KN‐93 in adjunct with nano‐C60 for OS therapy. Considering that CaMKIIα is important for tumor progression in several tumor types,^14^, ^15^, ^16^, ^17^ we speculate that CaMKIIα inhibition may improve the efficacy of nano‐C60 in other tumors with high expression of CaMKIIα as well. Further studies are required to address these possibilities.

Under normal physiological conditions, autophagy occurs at a relatively low basal level, playing a cytoprotective role by maintaining cellular homeostasis. During tumor development, elevated levels of autophagy play paradoxical roles in promoting both cell survival and cell death.^45^ Recent studies demonstrated that fullerene C60 and its derivatives act as autophagy activators that sensitize cancer cells to chemotherapeutic killing.^21^, ^27^ Nevertheless, in these studies, only autophagosome accumulation was shown, with no autophagic flux investigated. In the current work, we examined the autophagic response elicited by nano‐C60 in detail and discovered that nano‐C60 induced an increase in autophagosome formation and a decrease in autophagic turnover.

Interestingly, inhibition of CaMKIIα further promoted nano‐C60‐induced autophagy. Currently, only a few groups have shown the involvement of CaMKII in autophagy initiation. For example, inhibition of CaMKIIγ has been shown to trigger apoptosis and autophagy in colorectal cancer cells.^46^ Nevertheless, the detailed influence of CaMKII on autophagic degradation has not been reported. Herein, we further demonstrated that inhibition of CaMKII by KN‐93 in nano‐C60‐treated cells enhanced autophagosome and SQSTM1/P62 accumulation, suggesting the ability of KN‐93 to promote incomplete autophagy induced by nano‐C60. Unexpectedly, Zhong et al. showed that activation of CaMKII was required to initiate autophagy in response to free fatty acids, and this activation was significantly inhibited by KN‐93 in a cardiac remodeling model.^47^ In human muscle cells, CaMKII inactivation by obestatin signaling contributed to the inhibition of FoxO‐dependent autophagy.^48^ In these findings, the relationship between CaMKII and autophagy conflicted with our current study. It is conceivable that CaMKII has diverse effects on autophagy by sensing various upstream signals and acting on distinct downstream targets in different cells and conditions.

Lysosomes, as the major intracellular degradation organelle, play an essential role in the degradation of aggregated or malfunctional proteins (e.g., via autophagy).^49^ Thus, lysosomal dysfunction has serious physiological and pathological consequences.^40^ In this study, we found for the first time that inhibition of CaMKII promoted lysosomal alkalinization, enlargement, and vacuolization, whereas nano‐C60 had no significant effect on lysosomal acidity. It is well documented that defective lysosomal degradation can induce lysosomal enlargement.^40^ Therefore, we speculate that KN‐93 disrupts the lysosomal degradation capacity by altering the acidic environment, thus enhancing nano‐C60‐induced autophagic flux inhibition. Moreover, blocking autophagic degradation further increases lysosomal enlargement and vacuolization. Thus, it is reasonable to speculate that the biological effects of KN‐93 and nano‐C60 on lysosomes reciprocally influence each other. Combining KN‐93 with nano‐C60 synergistically enhances lysosomal dysfunction and autophagic turnover inhibition. Nevertheless, the exact molecular mechanism of KN‐93 action on lysosomes remains undetermined.

One intriguing finding in the present study is that the downstream autophagy inhibitor HCQ enhanced the cytotoxicity induced by nano‐C60 and KN‐93 combination treatment, while the upstream autophagy inhibitor Wort had the opposite effect. These results indicated a correlation between excessive autophagosome accumulation and cell death. Since autophagy is now under investigation to enhance the efficacy of antitumor therapy, it is important to notice the different role of autophagy in tumor therapy. Our data provided a model that precise control of autophagy could maximize the antitumor effect of nanomaterials.

Phosphatidylinositol 3‐kinases (PtdIns3Ks) and phosphoinositide 3‐kinases (PI3Ks) are all involved in the autophagic process. As we all know, Wort blocks autophagosome formation via suppression of the class III PtdIns3K pathway, which is a positive regulator of autophagy.^50^ However, PI3‐Kinases have their ATP binding sites at the kinase domain in common since they are evolutionarily related. Therefore, Wort was also found to interfere with the activity of class I PI3‐Kinase by blocking the ATP binding site. Class I PI3‐Kinase inhibits autophagy through the well‐documented PI3K‐AKT‐mTOR complex 1 (MTORC1) pathway and involves in cell survival, metabolism, migration, etc.^50^ Thus, Wort inhibits autophagy only in a certain “pharmacological window.” In our study, Wort effectively inhibited LC3‐II conversion induced by nano‐C60 alone (Figure S5A, Supporting Information) or nano‐C60 and KN‐93 co‐treatment (Figure 6C); nevertheless, there was no obvious effect on the class I PI3K‐AKT signaling (Figure S8, Supporting Information). These results eliminated the interference of Wort on class I PI3K‐AKT signaling and demonstrated that Wort at the concentration we used was able to inhibit autophagy.

Another interesting point is that nano‐C60 requires photoactivation for ROS production.^18^, ^21^ This requirement appears to be minimal, as normal laboratory light exposure during cell handling was sufficient to induce ROS production. Nonetheless, as minimal light exposure cannot be achieved in most in vivo conditions, the cytotoxicity induced by nano‐C60 alone may be weaker in vivo than in vitro. Therefore, in addition to photoactivation, the antitumor effect of nano‐C60 should be improved through other methods such as drug combination.

Collectively, this report demonstrates the antagonism of nano‐C60‐CaMKIIα interactions in nano‐C60‐induced antitumor effect. CaMKIIα inhibition sensitizes OS cells to nano‐C60 through suppression of autophagic degradation. So, these findings suggest that CaMKIIα activity should be considered when employing nano‐C60 for tumor therapy. Therefore, our study provides new guidance for the rational design and synthesis of nano‐C60‐based antitumor nanomedicines. For instance, a new nanoplatform based on nano‐C60 can be designed to combine CaMKIIα inhibition, autophagy modulation, with other strategies such as photodynamic therapy for augmenting nano‐C60‐elicited antitumor capacity.

## 4 Conclusion

In summary, we confirmed the biological effect of nano‐C60−CaMKIIα interactions on tumor therapy and investigated the relationship between CaMKIIα inhibition and autophagic degradation. In addition to its direct cytotoxic effect via ROS production, nano‐C60 was found to have a strong ability to induce autonomous CaMKIIα activity in OS cells. Nevertheless, CaMKIIα activation played a cytoprotective role to counteract nano‐C60‐induced cytotoxicity. Inhibition of CaMKIIα activity by either the chemical inhibitor KN‐93 or CaMKIIα knockdown significantly enhanced the antitumor effect of nano‐C60 in vitro and in vivo. Mechanistically, CaMKIIα inhibition caused lysosomal dysfunction through lysosomal alkalinization, which inhibited autophagic degradation and promoted abnormal autophagosome accumulation, leading to an increase in nano‐C60‐induced cytotoxicity in OS cells. Importantly, the downstream autophagy inhibitor HCQ enhanced cell death after nano‐C60 and KN‐93 co‐treatment, whereas the upstream autophagy inhibitor Wort has the opposite effect. These results indicated that excessive autophagosome accumulation and autophagic degradation blocking play an important role in KN‐93‐enhanced‐OS cell death. This data provides a model demonstrating that precise control of autophagy maximizes the antitumor effect of nanomaterials. Therefore, inhibiting CaMKIIα activity renders OS cells more vulnerable to nano‐C60 by suppressing autophagic degradation, which may represent a novel and effective strategy for improving the efficacy of nano‐C60 in antitumor therapy.

## 5 Experimental Section

*Materials*: Ultrapure water (pH 6.7; Milli‐Q, Bedford, MA, USA) was used for all experimental conditions. Fullerene C60 (99.9% pure) and KN‐93 (Cat. No. K1385) were purchased from Sigma‐Aldrich. Bovine CaM (Cat. No. 208 690) was purchased from Calbiochem. Wort (Cat. No. S2758) and HCQ (Cat. No. S4430) were purchased from Selleckchem. Antibodies against CaMKII (Cat. No. ab52476) and phospho‐T286 CaMKII (Cat. No. ab32678) were purchased from Abcam. Anti‐LC3 antibody (NB100‐2220) was purchased from Novus Biologicals. Antibodies against SQSTM1/p62 (39749), phospho‐AKT (4060), total AKT (4691), cathepsin B (31718), cathepsin D (2284) were purchased from Cell Signaling Technology. Anti‐GAPDH antibody (Cat. No. MAB374) was purchased from Millipore. Alexa Fluor 594 AffiniPure Goat anti‐rabbit IgG was purchased from Jackson ImmunoResearch Laboratories.

*CaMKIIα Autophosphorylation*: Briefly, 143B cell lysates were pretreated with 500.0 × 10^−6^ m CaCl_2_, 1.0 × 10^−6^ m CaM, and 10.0 × 10^−3^ m MgCl_2_ for 1 min on ice, and 4.0 µg mL^−1^ of nano‐C60 was subsequently added and then incubated for 20 min, followed by the addition of 1.0 × 10^−3^ m EGTA for another 10 min on ice. CaMKIIα phosphorylation was triggered by treatment with 1.0 × 10^−3^ m ATP and terminated with sodium dodecyl sulfate (SDS) loading buffer after 30 min. CaMKIIα autophosphorylation at residue T286 was measured by Western blotting with the anti‐phospho‐CaMKII antibody.

*Cell Death Assay*: Cells were treated with 10.0 × 10^−3^ m Hoechst 33342 and 10.0 × 10^−3^ m PI for 20 min. After washing with PBS three times, cells were detected by fluorescence microscopy (Leica, Wetzlar, Germany). Cell death was quantified by counting at least 400 cells per group and was expressed as the ratio of PI‐positive to Hoechst 33 342‐positive cells. These assays were independently completed by two of the authors in a double‐blind manner.

*CaMKIIα siRNA Transfection*: For siRNA transfection, 143B cells were cultured in 6‐well plates. After 12 h, the cells were transfected with 20.0 × 10^−9^ m of CaMKIIα siRNA (sc‐29 900, Santa Cruz Biotechnology, Santa Cruz, CA, USA) per well using Lipofectamine 2000 (Invitrogen, USA) and following the manufacturer's protocol.

*EGFP‐LC3 Puncta Formation Assay*: EGFP‐LC3 puncta was quantified by counting at least 200 cells and demonstrated as the percentage of EGFP‐LC3‐positive cells (cells with at least five EGFP‐LC3 dots). The assays were independently completed in a double‐blind manner.

*Lysosomal Acidity Assay*: 143B cells were treated with 1.0 µg mL^−1^ nano‐C60 in the presence or absence of 7.5 × 10^−6^ m KN‐93 for 12 h and were then harvested and washed twice in PBS. After incubating with 500.0 µL of prewarmed medium containing 1.0 × 10^−6^ m LysoSensor Green DND‐189 dye (L‐7535, Invitrogen, USA) for 30 min, cells were washed and resuspended with PBS. Lysosomal acidity was analyzed by flow cytometry (FACSCalibur, BD, San Jose, CA, USA).

*DQ‐BSA Assay*: MG63‐EGFP‐LC3 cells were incubated in DMEM containing DQ Red BSA (D‐12051, Molecular Probes) at a final concentration of 10.0 µg mL^−1^ for 3 h at 37 °C, washed twice with PBS and imaged by fluorescence microscopy (Leica, Wetzlar, Germany). The number of the fluorescent fragments in each cell was quantified, and at least 60 cells were included for each group.

*Animals and Tumor Models*: Male BALB/c nude mice (6−8 weeks) were purchased from Shanghai Slac Laboratory Animal Co., Ltd. (Shanghai, China). Next, 143B tumor cells (1.0 × 10^6^) resuspended in 100.0 µL of cold PBS were inoculated into the right flank of each nude mouse. After 1 week, mice were randomly divided into four groups (five mice in each group): PBS, nano‐C60 alone, KN‐93 alone, and nano‐C60+KN‐93. Nano‐C60 (0.2 mg kg BW^−1^) and KN‐93 (0.5 mg kg BW^−1^) were subcutaneously (s.c.) injected into the tumor every 2 days. Body weight and tumor sizes were also measured every 2 days. Tumor volume was estimated using the following equation:

$$\mathrm{Tumor}\;\mathrm{Volume}=\frac{\mathrm{Length}\times {\mathrm{Width}}^{2}}{2}$$

After 14 days, all mice were sacrificed, and tumors were removed and weighed. All animal care and experimental procedures were approved by the Animal Care and Use Committee of Shanghai General Hospital.

*TUNEL Assay*: Apoptosis was detected using an In Situ Cell Death Detection Kit, Fluorescein (Roche Diagnostics, Mannheim, Germany). Paraffin‐embedded 143B tumor sections were deparaffinized using xylene and ethanol. Then, the sections were rehydrated by addition of proteinase K at room temperature for 3 min and incubation with equilibration buffer for 30 min. The TUNEL reaction mixture was added to the sections and then incubated at 37 °C for another 60 min. After washing twice with PBS, the sections were stained with DAPI, and randomly chosen fields were observed under a fluorescence microscope (Leica, Wetzlar, Germany).

*Statistical Analysis*: All data were presented as the mean ± SEM and were analyzed by two‐tailed Student's *t*‐tests. *P* < 0.05 was considered significant, and *P* < 0.01 and *P* < 0.005 were highly significant.

## Conflict of Interest

The authors declare no conflict of interest.

## Supporting information

Supplementary
