# Supplementary material for: Inhibition of CaMKIIα Activity Enhances Antitumor Effect of Fullerene C60 Nanocrystals by Suppression of Autophagic Degradation
Source: Adv Sci (Weinh). 2019 Feb 10;6(8):1801233. doi: 10.1002/advs.201801233 (PMC6468974; doi:10.1002/advs.201801233)
Supplement: Supplementary file 1 — Supplementary [file ADVS-6-1801233-s001.pdf]

Copyright WILEY-VCH Verlag GmbH & Co. KGaA, 69469 Weinheim, Germany, 2019.

ADVANCED SCIENCE
Open Access

Supporting Information

for *Adv. Sci.,* DOI: 10.1002/advs.201801233

Inhibition of CaMKII# Activity Enhances Antitumor Effect of Fullerene C60 Nanocrystals by Suppression of Autophagic Degradation

Jing Xu, Hongsheng Wang, Yi Hu, Yu Shrike Zhang, Longping Wen, Fei Yin, Zhuoying Wang, Yingchao Zhang, Suoyuan Li, Yanyan Miao, Binhui Lin, Dongqing Zuo, Gangyang Wang, Min Mao, Tao Zhang, Jianxun Ding, Yingqi Hua,* and Zhengdong Cai**

Copyright WILEY-VCH Verlag GmbH & Co. KGaA, 69469 Weinheim, Germany, 2019.

Supporting Information

Inhibition of CaMKIIα Activity Enhances Antitumor Effect of Fullerene C60 Nanocrystals by Suppression of Autophagic Degradation

Jing Xu, Hongsheng Wang, Yi Hu, Yu Shrike Zhang, Longping Wen, Fei Yin, Zhuoying Wang, Yingchao Zhang, Suoyuan Li, Yanyan Miao, Binhui Lin, Dongqing Zuo, Gangyang Wang, Min Mao, Tao Zhang, Jianxun Ding, Yingqi Hua,* and Zhengdong Cai**

Dr. J. Xu, F. Yin, Z. Wang, Dr. T. Zhang, Prof. Y. Hua, Prof. Z. Cai
Department of Orthopedics, Shanghai General Hospital, Shanghai Jiao Tong University School of Medicine
Shanghai Bone Tumor Institution
100 Haining Street, Shanghai 200080, P. R. China
E-mail: hua_yingqi@163.com; caizhengdong@sjtu.edu.cn
Prof. J. Ding
Key Laboratory of Polymer Ecomaterials, Changchun Institute of Applied Chemistry, Chinese Academy of Sciences
5625 Renmin Street, Changchun 130022, P. R. China
E-mail: jxding@ciac.ac.cn
Prof. Y. S. Zhang
Division of Engineering in Medicine, Department of Medicine, Brigham and Women's Hospital, Harvard Medical School
65 Landsdowne Street, Cambridge, MA 02139, USA
Prof. L. Wen
School of Medicine, South China University of Technology
Nanobio Laboratory, Institutes for Life Sciences, South China University of Technology
381 Wushan Street, Guangzhou 510006, P. R. China
Dr. H. Wang, Y. Zhang, S. Li, B. Lin, Dr. D. Zuo, Dr. G. Wang, M. Min
Shanghai Bone Tumor Institution
100 Haining Street, Shanghai 200080, P. R. China
Y. Hu
Hefei National Laboratory for Physical Sciences at the Microscale and School of Life Sciences, University of Science and Technology of China
96 Jinzhai Street, Hefei 230026, P. R. China
Dr. Y. Miao
Key Laboratory of Gene Engineering of the Ministry of Education, State Key Laboratory of Biocontrol, School of Life Sciences, Sun Yat-sen University
135 West Xingang Street, Guangzhou 510275, P. R. China

Experiment Section

Nanoparticles Preparation: Water-suspended nano-C60 was prepared as previously described in our previous study.[1] Briefly, 10.0 mg of fullerene C60 was added to 500.0 mL of tetrahydrofuran (THF), which was previously unopened, and then stirred in the dark for 72 h. The insoluble fullerene C60 was removed using filtration. Subsequently, 500.0 mL of ultrapure water was added to the fullerene C60 solution over a span of 30 s, followed by removal of THF using a rotary evaporator (BC-R202, Shanghai Biochemical Equipment Co., Shanghai, P. R. China). The mixture was gently heated to approximately 75 − 80 °C, and the final volume was approximately 50.0 mL.

Cell Viability Assay: Cell viability was determined using cholecystokinin octapeptide (CCK-8; Dojindo, Kumamoto, Japan) assays. Cell suspensions were seeded into 96-well plates at a density of approximately 1.0×10^4 cells per well and cultured overnight. After different treatments, 10.0 μL of CCK-8 solution was added to each well and then incubated at 37 °C for 2 h. The absorbance was measured at 450 nm using a spectrophotometer (Thermo Scientific, USA).

Measurement of ROS: Generation of intracellular reactive oxygen species (ROS) was measured using dihydroethidium (DHE; S0063, Beyotime, Shanghai, P. R. China), according to the instructions of manufacturer. In brief, cells were seeded in 6-well plates at a density of 2.0×10^5 cells/well. After different treatment, the cells were incubated with DHE (10.0 μM) for 30 min at 37 °C. The level of ROS was assessed by fluorescence microscopy (Leica, Wetzlar, Germany).

Establishing MG63-EGFP-LC3 and 143B-RFP-Lamp1 Cell Lines: Cells were transfected with EGFP-LC3 or RFP-LAMP1 plasmid using Lipofectamine 2000 according to the protocol of manufacturer. Twenty-four hours post-transfection, cells were cultured in Dulbecco's modified Eagle medium (DMEM) containing 0.6 mg mL^{-1} of G418 (Promega, USA). Then,

after approximately 20 days, cell colonies with strong green or red fluorescence were selected under a fluorescence microscope (Leica, Wetzlar, Germany).

[1] Q. Zhang, W. Yang, N. Man, F. Zheng, Y. Shen, K. Sun, Y. Li, L. P. Wen, *Autophagy* **2009**, *5*, 1107.

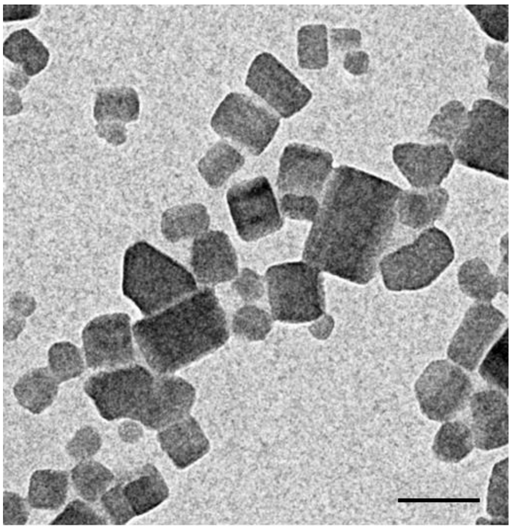

Figure S1. Transmission electron microscopy (TEM) observation. The water-suspended nano-C60 was determined by TEM. Scale bar = 100.0 nm.

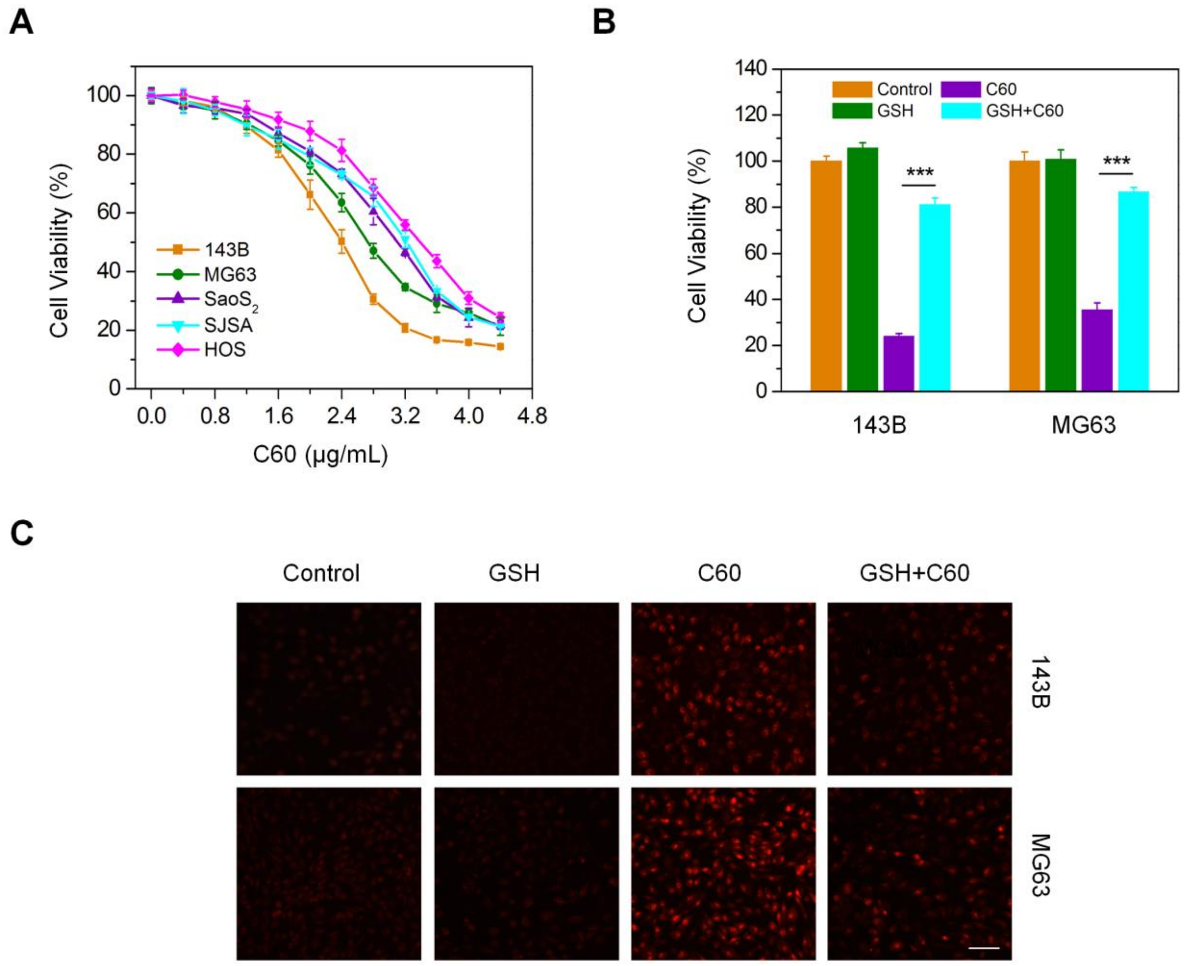

Figure S2. Cytotoxicity of nano-C60 by ROS production in osteosarcoma cells. **A**) 143B, MG63, $SaoS_2$, SJSA, and HOS cells were treated with various concentrations of nano-C60 for 24 h. The cell viability was assessed by CCK-8 assay. Mean ± SEM, *n* = 3. **B**) 143B and MG63 cells were treated with 3.2 μg mL^{-1} nano-C60 for 24 h in the presence or absence of 1.0 mM GSH. The cell viability was determined by CCK-8 assay. The cell viability was determined by CCK-8 assay. Mean ± SEM, *n* = 3. ****P* < 0.005. **C**) 143B and MG63 cells were treated with 1.6 μg mL^{-1} nano-C60 for 12 h in the presence or absence of 1.0 mM GSH, and then loaded with DHE for 30 min. The level of ROS was detected by fluorescence microscopy. Representative images were presented. Scale bar = 100.0 μm

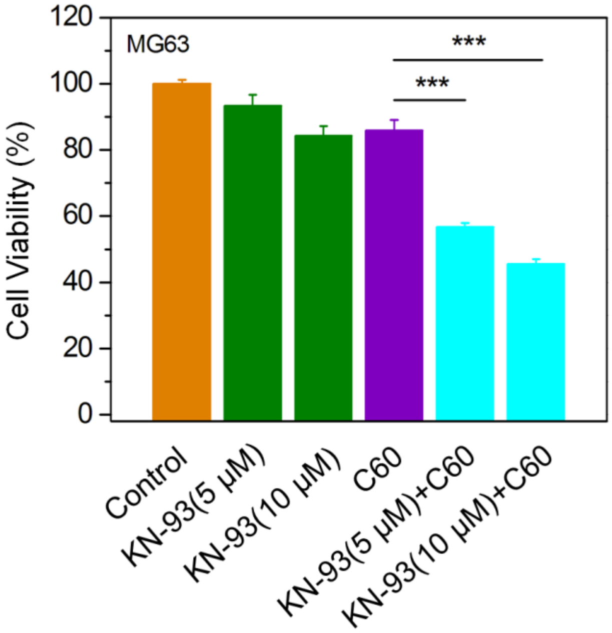

Figure S3. Cell viability of MG63 cells treated with KN-93 and nano-C60. MG63 cells were treated with or without 1.6 μg mL^{-1} nano-C60 in the presence or absence of 5.0 μM or 10.0 μM KN-93 for 24 h. Cell viability was determined by CCK-8 assay. Mean ± SEM, $n = 3$. ***$P < 0.005$.

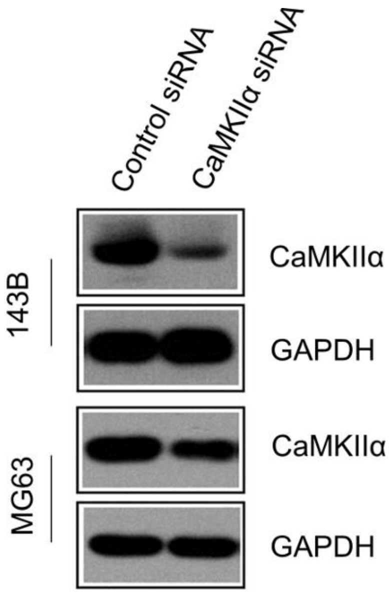

Figure S4. Western blotting of CaMKIIα in 143B and MG63 cells transfected with CaMKIIα siRNA or control siRNA for 48 h.

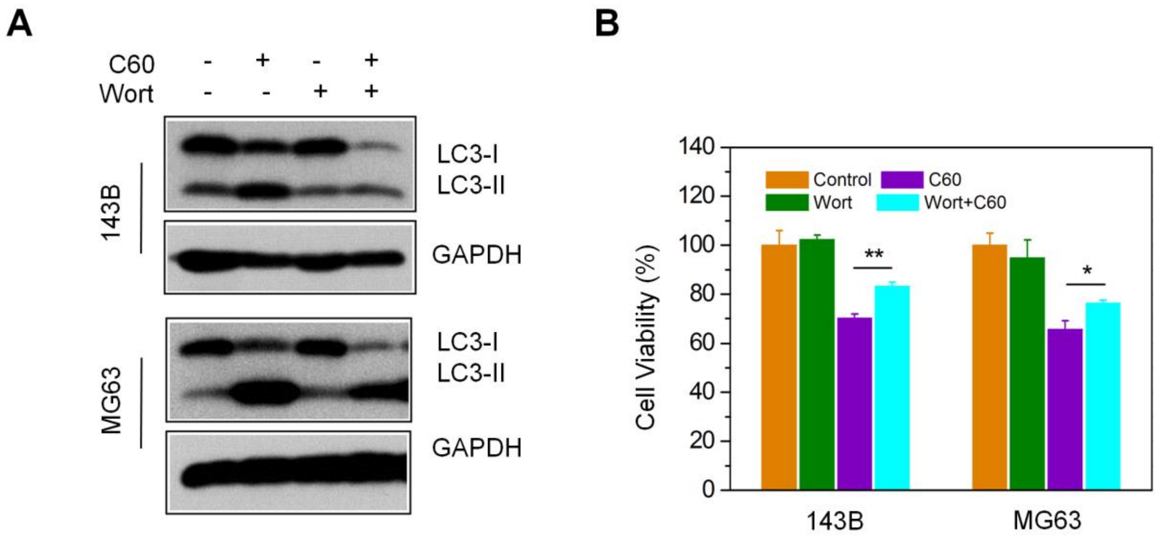

Figure S5. Effect of wortmannin (Wort) in nano-C60-treated OS cells. **A**) 143B and MG63 cells were treated with or without 1.6 μg mL^{-1} nano-C60 in the presence or absence of Wort for 24 h. **B**) 143B and MG63 cells were treated as described in **A**). The cell viability was assessed by CCK-8 assay. Mean ± SEM, *n* = 3. **P* < 0.05, ***P* < 0.01.

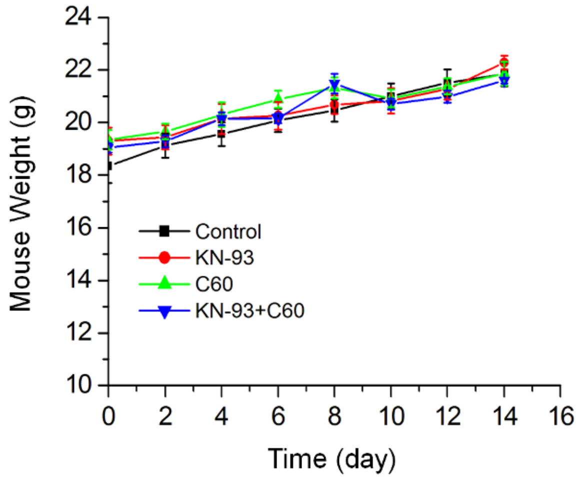

Figure S6. Body weights for each group were measured every two days.

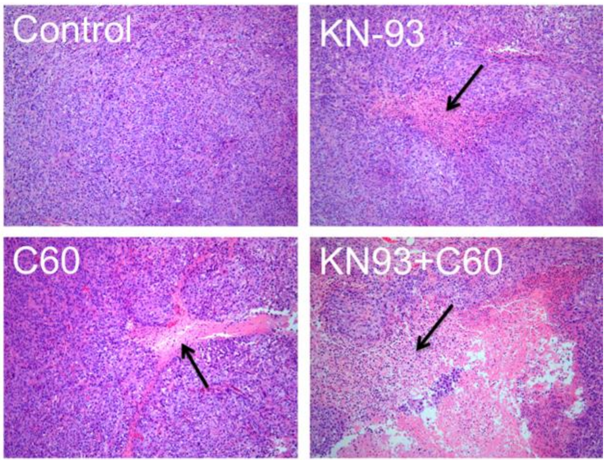

Figure S7. H&E analysis of tumor tissues for each group. The black arrows indicated necrotic tissues.

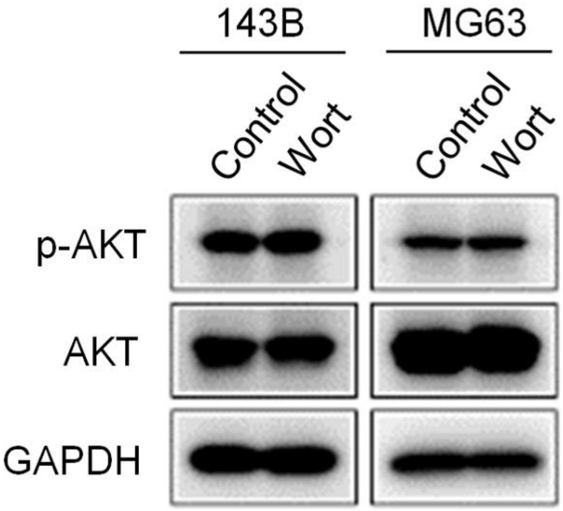

Figure S8. Western blot analysis of pi-AKT levels of 143B and MG63 cells treated with 100.0 nM Wort.
